# Aortic Valve Infective Endocarditis with Root Abscess: Root Repair Versus Root Replacement

**DOI:** 10.3390/pathogens14070626

**Published:** 2025-06-23

**Authors:** Zaki Haidari, Stephan Knipp, Iskandar Turaev, Mohamed El Gabry

**Affiliations:** Department of Thoracic and Cardiovascular Surgery, West German Heart and Vascular Center Essen, University Hospital Essen, 55 45147 Essen, Germanyiskandar.jamoliddin@gmail.com (I.T.); mohamed.elgabry@uk-essen.de (M.E.G.)

**Keywords:** infective endocarditis, aortic valve, root abscess, repair, replacement

## Abstract

Background: Aortic valve infective endocarditis (IE) complicated by an aortic root abscess is a challenging problem that leads to increased morbidity and mortality. Aortic root repair or replacement are two potential treatment options. We aimed to compare patients undergoing aortic root repair or replacement with short- and mid-term outcomes. Methods: Consecutive patients with active aortic valve IE complicated by aortic root abscess undergoing cardiac surgery from January 2012 to January 2022 were included. Patients receiving aortic root repair were compared to patients undergoing aortic root replacement. Endpoints included overall mortality, incidence of recurrent IE and re-intervention during a two-year follow-up period. Inverse propensity weighting was employed to adjust for confounders. Results: Seventy-three patients with aortic valve IE with root abscess underwent surgical therapy. Fifty-six patients received aortic root repair and seventeen patients underwent aortic root replacement. Patients undergoing root replacement had significantly higher surgical risk (EuroSCORE II: 9 versus 19, *p* = 0.02) and extended disease (circumferential annular abscess: 9% versus 41%, *p* < 0.01). Inverse propensity weighted analysis revealed no relationship between surgical strategy and outcome. Weighted regression analysis revealed EuroSCORE II and disease extension as significant predictors of 30-day and 2-year mortality. Conclusions: In patients with aortic valve IE with root abscess, root repair is mostly performed in lower-risk patients with limited disease extension. Short- and mid-term mortality, recurrent endocarditis and reintervention were comparable between surgical strategies during follow-up. Surgical risk and disease extension, rather than surgical strategy, seem to be significant predictors of short- and mid-term mortality.

## 1. Introduction

Aortic valve infective endocarditis (IE) complicated by aortic root abscess is associated with increased mortality and morbidity [1,2,3,4,5,6,7,8]. Radical surgical debridement of the abscess cavity is necessary to prevent recurrent infective endocarditis [9,10]. However, extensive surgical debridement often results in tissue defects of the aortic root, leading to the necessity for root repair or replacement. Current guidelines discourage patch reconstruction and recommend root replacement to avoid recurrence, leaks or pseudoaneurysm formation [11]. Despite this, detailed data comparing root repair with root replacement in these high-risk patients are scarce. In the present study, we therefore aimed to compare the concept of aortic root repair with aortic root replacement on clinical outcomes in patients with active aortic valve IE complicated by root abscess undergoing surgical therapy.

## 2. Methods

### 2.1. Patients

From January 2012 to January 2022, patients with proven IE [12] of the aortic valve complicated by root abscess undergoing cardiac surgery were included. Patient data were prospectively recorded and retrospectively extracted. An aortic root abscess was defined as either the paravalvular region of the aortic valve with purulent or necrotic tissue with or without fistula or as aorto-ventricular discontinuity. Patients with previous aortic root replacement were excluded as were patients undergoing replacement of the intervalvular fibrous body due to the high complexity and invasiveness of the operation [13].

### 2.2. Surgical Technique

**Figure 1:**
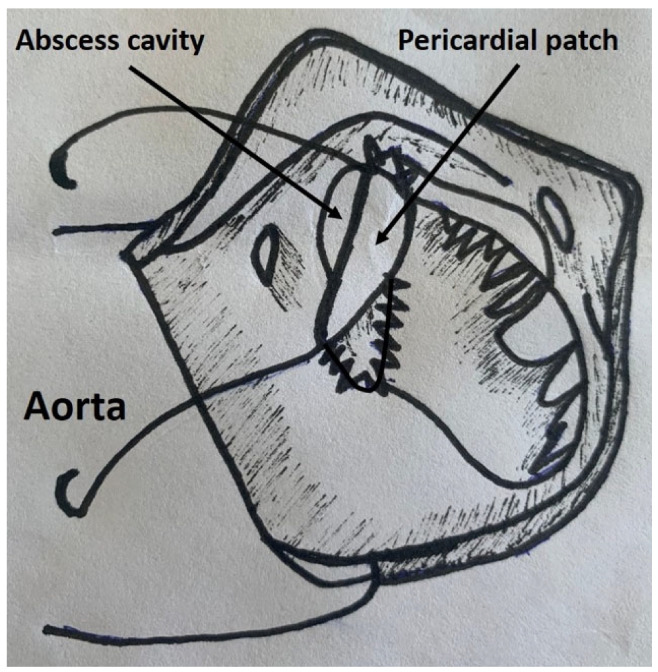
Aortic root repair after radical surgical debridement of the abscess cavity.

All surgical procedures were performed via median sternotomy. Cardiopulmonary bypass was established by standard aortic and caval cannulation techniques. Cold crystalloid cardioplegia (Custodiol, Dr. Franz Koehler Chemie, Bensheim, Germany) was used for myocardial protection. After aortotomy and resection of the aortic valve leaflets, the aortic root was inspected for the presence of abscesses. In all cases, drainage and radical surgical debridement of the abscess cavity was performed. After disinfection with diluted povidone–iodine solution, a decision was made to either repair or replace the aortic root. The decision was dependent on several factors, such as the extension of the defect and experience and preference of the operating surgeon. In patients with abscesses limited to one or two cusps, root repair (Figure 1) was preferred. On the other hand, root replacement was performed in cases with prosthesis endocarditis and/or extended/circumferential abscesses. Furthermore, patients with previous rapid deployment valve prosthesis or transcatheter aortic valves often required root replacement due to extensive root destruction. In cases with friable annular tissue, a strip of pericardial patch was used to reinforce the annulus. In cases of root repair, a pericardial patch was used to exclude the defect. Before the closure of the patch, a mixture of antibiotic (this was dependent on the causative microorganism but the antibiotic used was usually vancomycin) and fibrin glue was considered to fill the remaining abscess cavity. The aortic valve was replaced by a stented aortic valve prosthesis. In cases selected for root replacement, a Bentall procedure was performed. Concomitant coronary artery bypass grafting and/or additional valvular procedures were performed whenever indicated. All instruments, synthetic material and prosthesis were soaked in a vancomycin solution during the operation. Postoperatively, all patients were transferred to the cardiac surgical intensive care unit. Postoperative care consisted of invasive hemodynamic monitoring and guideline-directed antibiotic and supportive therapy.

### 2.3. Outcome Measures

The primary endpoints of the study were the incidence of recurrent IE, reoperation and mortality during a two-year follow-up period. Recurrent IE was defined as relapse (with the same causative microorganism as the index operation) or reinfection (with a new causative microorganism as the index operation) of the aortic valve prosthesis or aortic root. Secondary endpoints consisted of postoperative complications, organ failure requiring support (mechanical circulatory support, reintubation after extubation and new-onset dialysis) and intensive care unit and hospital stay.

### 2.4. Data Collection and Analysis

Perioperative characteristics were collected from the institutional electronic database. Data on survival and valve-related complications were assessed via outpatient visits or telephone follow-up. The data were analyzed by SPSS software version 30 (SPSS Inc., Chicago, IL, USA). Categorical variables were reported as numbers and compared using the χ^2^ test or Fisher’s exact test. Continuous variables were expressed as median and interquartile range and compared using the Mann–Whitney test. The Shapiro–Wilk test was used to test the normality of the data.

Standardized mean differences (SMD) of preoperative variables were calculated to assess balances between the two groups. An SMD value < 0.1 was considered an acceptable mean or frequency difference between the repair and replacement groups. Inverse propensity weighting (IPW) was performed to correct for imbalances between the two comparing groups. The IPW model included age, sex, previous cardiac surgery, EuroSCORE II, surgical delay and indication for surgery. Weighted logistic regression was employed to compare outcomes between the groups. A *p*-value < 0.05 was considered statistically significant.

## 3. Results

### 3.1. Baseline Characteristics

Over a ten-year period, a total of 79 consecutive patients underwent cardiac surgery for IE of the aortic valve in combination with aortic root abscess. Six patients were excluded: three patients had previous aortic root replacement and three patients underwent replacement of the intervalvular fibrous body. Aortic root repair and root replacement was performed in 56 and 17 patients, respectively. Baseline demographics and clinical and inflammatory status of the two groups are shown in Table 1. Sixty-two percent of the patients had prior cardiac surgery. Prosthetic aortic valve IE was present in 43 (59%) of cases: 54% in the repair group and 76% in the replacement group (*p* = 0.16). The EuroSCORE II was significantly higher in the root replacement group. There were no statistically significant differences in other demographics, hemodynamic and pulmonary status, or the levels of preoperative inflammatory parameters between both groups. Staphylococcal species were the most commonly identified causative microorganisms for the IE. The indication for surgery was mostly preoperatively identified uncontrolled infection, which was significantly more common in the replacement group (52% vs. 82%, *p* = 0.03). However, intraoperative identification of root abscess was more frequent in the root repair group (Table 2).

### 3.2. Operative Characteristics

**Figure 2:**
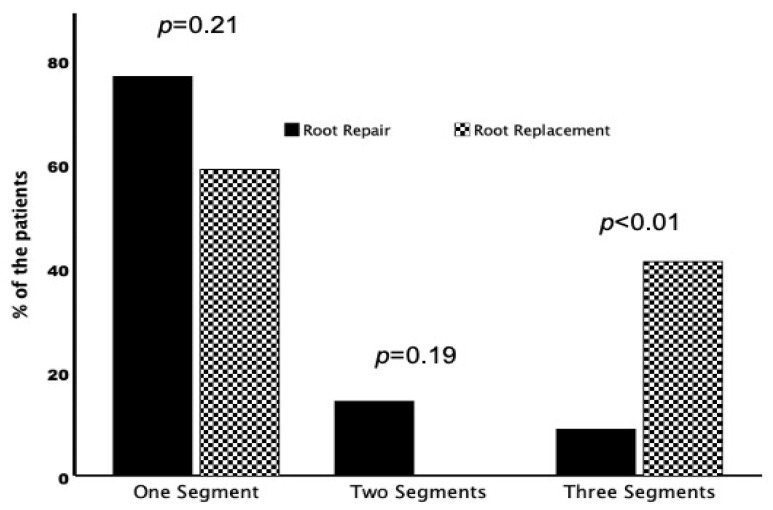
Number of involved segments in the aortic root abscess according to the procedure performed. One segment represents one-third of the area of the aortic root.

In patients undergoing root repair, the root abscess was mostly limited to one (one cusp/commissure) of the three segments of the aortic root (Figure 2). A circumferential abscess (three segements) was present in five (9%) patients from the root repair and in seven (41%) patients from the root replacement group (*p* < 0.01). Pericardial patch reconstruction was more often performed in patients undergoing root repair. In patients with small abscesses, direct closure of the defect was preferred. A stented biological valve prosthesis was implanted in almost all patients in the repair group. In the root replacement group, almost all patients were treated with a stentless xenograft aortic conduit. Concomitant coronary artery bypass grafting was more common in the root repair group and concomitant additional valvular procedures were performed more frequently in the root replacement group, without statistical significance. Cardiopulmonary bypass and aortic cross-clamp times were longer in the root replacement group but did not reach statistical significance (Table 3).

### 3.3. Endpoints

Follow-up was 100% complete. The crude endpoints and adjusted odds ratios (aOR) are summarized in Table 4. The overall thirty-day mortality was 22%, with 16% in the root repair group and 41% in the root replacement group. The causes of early mortality were septic multiorgan failure in ten cases (63%), heart failure in four cases (25%) and liver failure in one case (6%). Two-year mortality increased to 34%, with 29% in the root repair group and 53% in the root replacement group. After adjustment for confounders, surgical strategy was not associated with short- and mid-term mortality. The adjusted incidence of postoperative organ failure requiring (temporary) support or replacement and complications were similar between groups. During the two-year follow-up period, recurrent IE occurred in three (5%) patients (40 days, 7 months and 20 months after the index operation, respectively) in the root repair group and in one (6%) patient (2 months after the index operation) in the root replacement group (*p* > 0.99). The identified causative microorganisms of the recurrence patients at the time of the index operation were *Staphylococcus aureus* (three patients: two in the repair group and one in the replacement group) and *Streptococcus mutans* (one patient in the repair group). All three recurrent IE patients in the root repair group underwent reoperation. However, the patient with recurrent IE in the root replacement group was deemed inoperable due to high surgical risk.

### 3.4. Weighted Logistic Regression Analysis

Univariable weighted logistic regression analysis revealed age, history of previous cardiac surgery, EuroSCORE II and three involved segments (circumferential annular abscess) as significant predictors of 30-day and two-year mortality (Table 5). Surgical strategy (root repair versus root replacement) was not correlated to short- and mid-term mortality.

## 4. Discussion

Aortic root abscess is a frequent and potentially lethal complication of aortic valve IE. The optimal surgical approach to tackle this complication has been debated for decades. In the present study, two common surgical techniques (root repair and root replacement) were compared regarding short- and mid-term outcomes. The results showed that root repair was associated with better outcome in terms of short- and mid-term mortality. Furthermore, the risk of recurrent IE and reoperation were comparable between the two surgical strategies during the two-year follow-up. However, root repair was performed in patients with limited disease extension and low risk as assessed by the EuroSCORE II. After adjustments for these confounders, the surgical strategy did not affect the outcome. The surgical risk as defined by EuroSCORE II (including age and history of previous cardiac surgery) and disease extension (number of infected segments of the aortic root) were associated with 30-day and two-year mortality.

According to the current literature, about one-third of patients with aortic valve IE present with abscess of the aortic root. Abscess formation is a strong predictor of short-term mortality and recurrent IE [14]. The optimal surgical strategy in patients with aortic root abscess has been evaluated in several studies before. The most important surgical aspect in cases with aortic root abscess remains the strict eradication of all infected tissue, which consists of abscess drainage and radical debridement. The best substitute for the aortic root remains controversial. Several surgical approaches have been proposed, including conventional root repair with a patch and root replacement using different grafts [15].

Earlier studies showed a survival benefit for patients receiving root replacement compared to root repair in patients with aortic valve IE complicated with root abscesses [16,17,18]. However, a meta-analysis showed an increased risk of short-term mortality and a lower risk of one-year reoperation in root abscess patients undergoing aortic root replacement. Furthermore, most studies used a homograft in cases with root replacement, which are not always available and are associated with higher rates of structural valve degeneration and reoperation [19,20].

When comparing root repair to root replacement, head-to-head comparison studies are scarce. Small, non-randomized and biased studies show comparable outcomes between the two techniques [21,22,23]. This was also the case in our study. Gollmann-Tepeköylü et al. compared aortic root repair with aortic root replacement in patients with aortic root abscess. Of note, aortic root repair was associated with significantly favorable long-term overall and event-free survival and freedom from recurrent IE. Long-term freedom from reoperation did not differ between the groups. Aortic root repair was an independent predictor of event-free survival [24]. These results were in line with a previous study by Rouzé et al. [25].

Although not always possible, the primary goal of surgery in such high-risk patients is to reduce the cardiopulmonary bypass and aortic cross-clamping times, which are longer in patients undergoing root replacement compared to root repair. These differences can be explained by the time needed for the mobilization and reimplantation of the coronary buttons during the root replacement procedure. The most important causes of short-term mortality remain postoperative septic multiorgan failure and heart failure, which are associated with longer cardiopulmonary bypass and cross-clamp times. These arguments favor root repair over root replacement whenever possible. Therefore, we recommend root repair in localized abscesses whenever possible. In patients with extensive/circumferential abscesses, root replacement can be considered. One could argue that root replacement is necessary and the only meaningful surgical option in those patients presenting with a circumferential aortic root abscess. Of note, the circumferential extent of the aortic root abscess cavity in the replacement group within the present analysis might have been the underlying cause of the significantly higher rate of preoperative uncontrolled infection and higher mortality. Intraoperative identified abscesses were more often in the repair group, as most of them were probably not detectable by preoperative echocardiography due to their small sizes.

## 5. Limitations

Although most of the baseline demographics, clinical and inflammatory statuses, and operative characteristics of the two groups were comparable, bias cannot be ruled out due to the retrospective study design, small sample size and absence of randomization. Furthermore, we employed IPW to adjust for confounders. Large and randomized studies are needed to evaluate the role of surgical strategy in patients with aortic valve IE complicated by root abscess.

## 6. Future Directions

Surgical outcomes of infective endocarditis remain poor due to high early postoperative mortality, which is attributed to hemodynamic instability caused by postoperative sepsis. Improvement in early postoperative mortality should be the main concern in endocarditis surgery. Adjunctive therapies (pre-, intra- and postoperatively), such as goal-directed antibiotic therapy (included therapeutic drug monitoring), contemporary hemodynamic support strategies or hemoadsorption techniques should be considered.

## Figures and Tables

**Table 1:** Baseline demographics, clinical status and inflammatory parameters.

	Root Repair N = 56	Root Replacement N = 17	SMD	*p*-Value
**Demographics**				
Age, years	70 (57–76)	65 (56–70)	0.411	0.11
Male gender	52 (93)	14 (82)	0.151	0.34
Coronary artery disease	24 (43)	8 (47)	0.036	0.76
Dialysis dependent	3 (5)	1 (6)	0.010	>0.99
Previous cardiac surgery	32 (57)	13 (76)	0.168	0.15
EuroSCORE II	9 (4–17)	19 (11–40)	0.765	0.02
**Clinical status**				
NYHA III-IV	17 (30)	5 (29)	0.009	0.94
Intubated	6 (11)	2 (12)	0.014	>0.99
Vasopressor need	7 (13)	3 (18)	0.063	0.69
Lactate, mmol/L	0.8 (0.6–1.1)	0.8 (0.6–1.3)	0.031	0.82
Surgical delay, days	11 (5–23)	6 (2–10)	0.357	0.14
Preserved LVF	39 (69)	12 (71)	0.003	>0.99
**Inflammatory parameters**				
C-reactive protein, mg/dL	6.8 (2.9–12.6)	9.3 (4.8–13.6)	0.093	0.54
Procalcitonin, ng/mL	0.24 (0.1–0.6)	0.23 (0.1–0.6)	0.017	>0.99
White blood count, 10^9^/L	8.8 (6.6–13.7)	8.6 (7.7–14.3)	0.005	0.95

Data are presented as the number (%) or median (IQR) and standardized mean difference (SMD). EuroSCORE: European System for Cardiac Operative Risk Evaluation; NYHA: New York Heart Association.

**Table 2:** Preoperative microbiological profile and surgical indications.

	Root Repair N = 56	Root Replacement N = 17	SMD	*p*-Value
**Causative microorganism**				
Staphylococcal species	21 (38)	6 (35)	0.019	0.87
*Staphylococcus aureus*	19	6	0.012	0.92
*Streptococcal* species	9 (16)	3 (18)	0.018	>0.99
*Enterococcal* species	11 (20)	3 (18)	0.021	>0.99
Others	4 (7)	2 (12)	0.071	0.62
Negative culture	12 (21)	3 (18)	0.040	>0.99
**Indication for surgery**				
Heart failure	10 (18)	2 (12)	0.069	0.72
Embolism	15 (27)	1 (6)	0.214	0.10
Uncontrolled infection	29 (52)	14 (82)	0.263	0.03
Valve disease	2 (3)	-	0.092	>0.99

Data are presented as the number (%) and standardized mean difference (SMD).

**Table 3:** Operative characteristics.

	Root Repair N = 56	Root Replacement N = 17	*p*-Value
Patch reconstruction	37 (66)	3 (18)	<0.01
Aortic valve prosthesis			
Biological	55 (98)	-	
Mechanical	1 (2)	-	
Aortic valved conduit			
Biological	-	15 (88)	
Mechanical	-	2 (12)	
Concomitant CABG	8 (14)	1 (6)	0.68
Concomitant MV procedure	16 (29)	7 (41)	0.33
Concomitant TV procedure	5 (9)	4 (24)	0.20
CPB time, minutes	134 (114–178)	177 (120–258)	0.54
ACC time, minutes	97 (78–126)	128 (94–201)	0.24

Data are presented as the number (%) or median (IQR). CABG: coronary artery bypass grafting; MV: mitral valve; TV: tricuspid valve; CPB: cardiopulmonary bypass; ACC: aortic cross-clamping.

**Table 4:** Crude endpoints and adjusted odd ratios for postoperative outcomes.

	Root Repair N = 56	Root Replacement N = 17	aOR (95% CI)	*p*-Value
**Primary**				
30-Day mortality	9 (16)	7 (41)	0.85 (0.65–1.12)	0.28
Re-endocarditis (2-year)	3 (5)	1 (6)	1.05 (0.51–2.18)	>0.99
Reoperation (2-year)	3 (5)	-	0.62 (0.54–0.71)	0.30
2-Year mortality	16 (29)	9 (53)	0.82 (0.63–1.07)	0.16
**Secondary**				
Myocardial infarction	1 (2)	1 (6)	1.72 (0.11–27.44)	0.70
Postoperative MCS	11 (20)	7 (41)	1.94 (0.80–4.73)	0.14
Dialysis	10 (18)	7 (41)	1.58 (0.69–3.62)	0.28
Reintubation	11 (20)	7 (41)	1.71 (0.72–4.09)	0.23
Resternotomy	4 (7)	5 (29)	2.54 (0.73–8.92)	0.15
Hospital stay, days	14 (8–18)	12 (6–18)	1.02 (0.99–1.05)	0.27

Data are presented as the number (%) or median (IQR) and adjusted odds ratio (aOR) with a 95% confidence interval (95% CI). MCS: mechanical circulatory support.

**Table 5:** Logistic regression analysis for predictors of 30-day and 2-year mortality.

	30-Day Mortality			2-Year Mortality		
Variable	aOR	95% CI	*p*	aOR	95% CI	*p*
Age, per year	1.13	1.08–1.19	<0.01	1.08	1.04–1.12	<0.01
Sex, female	1.84	0.42–8.12	0.42	1.16	0.26–5.07	0.85
Previous cardiac surgery	12.11	3.69–39.75	<0.01	6.29	2.66–14.88	<0.01
Surgical delay, days	0.98	0.95–1.01	0.25	0.99	0.96–1.01	0.35
EuroSCORE II, per point	1.08	1.05–1.11	<0.01	1.06	1.04–1.08	<0.01
Three involved segments	15.86	5.86–39.00	<0.01	9.89	3.90–25.09	<0.01
Surgical strategy, root repair	0.65	0.29–1.46	0.30	0.57	0.27–1.23	0.15

Data are presented as the adjusted odds ratio (aOR) and a 95% confidence interval (CI). EuroSCORE: European System for Cardiac Operative Risk Evaluation.

## Data Availability

The data presented in this study are available on request from the corresponding author. The data are not publicly available due to privacy or ethical restrictions.

## References

[1] Habib G., Erba P.A., Iung B., Donal E., Cosyns B., Laroche C., Popescu B.A., Prendergast B., Tornos P., Sadeghpour A. (2019). Clinical presentation, aetiology and outcome of infective endocarditis. Results of the ESC-EORP EURO-ENDO (European infective endocarditis) registry: A prospective cohort study. Eur. Heart J..

[2] David T.E. (1997). Surgical management of aortic root abscess. J. Card. Surg..

[3] David T.E., Regesta T., Gavra G., Armstrong S., Maganti M.D. (2007). Surgical treatment of paravalvular abscess: Long-term results. Eur. J. Cardiothorac. Surg..

[4] Leontyev S., Davierwala P.M., Krögh G., Feder S., Oberbach A., Bakhtiary F., Misfeld M., Borger M.A., Mohr F.W. (2016). Early and late outcomes of complex aortic root surgery in patients with aortic root abscesses. Eur. J. Cardio-Thorac. Surg..

[5] Watanabe G., Haverich A., Speier R., Dresler C., Borst H.G. (1994). Surgical treatment of active infective endocarditis with paravalvular involvement. J. Thorac. Cardiovasc. Surg..

[6] Danchin N., Retournay G., Stchepinsky O., Selton-Suty C., Voiriot P., Hoen B., Canton P., Villemot J.-P., Mathieu P., Cherrier F. (1999). Comparison of long term outcome in patients with or without aortic ring abscess treated surgically for aortic valve infective endocarditis. Heart.

[7] Langiulli M., Salomon P., Aronow W.S., McClung J.A., Belkin R.N. (2004). Comparison of outcomes in patients with active infective endocarditis with versus without paravalvular abscess and with and without valve replacement. Am. J. Cardiol..

[8] Castillo J.C., Anguita M.P., Ruiz M., Delgado M., Mesa D., Romo E., Arizón J.M., Vallés F. (2005). Clinical features and outcome of non-drug-addicted patients with infective endocarditis and perivalvular abscess. J. Heart Valve Dis..

[9] Aagaard J., Andersen P.V. (2001). Acute endocarditis treated with radical debridement and implantation of mechanical or stented bioprosthetic devices. Ann. Thorac. Surg..

[10] Yang B., Caceres J., Farhat L., Le T., Brown B., Pierre E.S., Wu X., Kim K.M., Patel H.J., Deeb G.M. (2021). Root abscess in the setting of infectious endocarditis: Short- and long-term outcomes. J. Thorac. Cardiovasc. Surg..

[11] Delgado V., Ajmone Marsan N., de Waha S., Bonaros N., Brida M., Burri H., Caselli S., Doenst T., Ederhy S., Erba P.A. (2023). 2023 ESC Guidelines for the management of endocarditis. Eur. Heart J..

[12] Li J.S., Sexton D.J., Mick N., Nettles R., Fowler V.G., Ryan T., Bashore T., Corey G.R. (2000). Proposed modifications to the Duke criteria for the diagnosis of infective endocarditis. Clin. Infect. Dis..

[13] Giambuzzi I., Bonalumi G., Di Mauro M., Roberto M., Corona S., Alamanni F., Zanobini M. (2021). Surgical aortic mitral curtain replacement: Systematic review and metanalysis of early and long-term results. J. Clin. Med..

[14] Ramanathan A., Witten J.C., Gordon S.M., Griffin B.P., Pettersson G.B., Shrestha N.K. (2021). Factors associated with local invasion in infective endocarditis: A nested case-control study. Clin. Microbiol. Infect..

[15] Smail H., Saxena P., Zimmet A.D., McGiffin D.C. (2016). Reconstruction and Replacement of the Aortic Root in Destructive Endocarditis. Oper. Tech. Thorac. Cardiovasc. Surg..

[16] Knosalla C., Weng Y., Yankah A.C., Siniawski H., Hofmeister J., Hammerschmidt R., Loebe M., Hetzer R. (2000). Surgical treatment of active infective aortic valve endocarditis with associated periannular abscess—11 year results. Eur. Heart J..

[17] Sabik J.F., Lytle B.W., Blackstone E.H., Marullo A.G., Pettersson G.B., Cosgrove D.M. (2002). Aortic root replacement with cryopreserved allograft for prosthetic valve endocarditis. Ann. Thorac. Surg..

[18] Yankah A.C., Pasic M., Klose H., Siniawski H., Weng Y., Hetzer R. (2005). Homograft reconstruction of the aortic root for endocarditis with periannular abscess: A 17-year study. Eur. J. Cardiothorac. Surg..

[19] Chen G.J., Lo W.C., Tseng H.W., Pan S.-C., Chen Y.-S., Chang S.-C. (2018). Outcome of surgical intervention for aortic root abscess: A meta-analysis. Eur. J. Cardiothorac. Surg..

[20] Harris W.M., Sinha S., Caputo M., Angelini G.D., A Vohra H. (2024). Surgical outcomes and optimal approach to treatment of aortic valve endocarditis with aortic root abscess—Systematic review and meta-analysis. Perfusion.

[21] Harris W.M., Sinha S., Caputo M., Angelini G.D., Ahmed E.M., Rajakaruna C., Benedetto U., Vohra H.A. (2022). Surgical outcomes and optimal approach to treatment of aortic valve endocarditis with aortic root abscess. J. Card. Surg..

[22] Elgalad A., Arafat A., Elshazly T., Elkahwagy M., Fawzy H., Wahby E., Taha A.-H., Sampaio L., Herregods M.-C., Peetermans W. (2019). Surgery for Active Infective Endocarditis of the Aortic Valve With Infection Extending Beyond the Leaflets. Heart Lung Circ..

[23] Elderia A., Wallau A.M., Bennour W., Gerfer S., Gaisendrees C., Krasivskyi I., Djordjevic I., Wahlers T., Weber C. (2024). Impact of Aortic Root Abscess on Surgical Outcomes of Infective Endocarditis. Life.

[24] Gollmann-Tepeköylü C., Abfalterer H., Pölzl L., Müller L., Grimm M., Holfeld J., Bonaros N., Bates K., Ulmer H., Ruttmann E. (2022). Impact of aortic root repair or replacement in severe destructive aortic valve endocarditis with paravalvular abscesses on long-term survival. Interact. Cardiovasc. Thorac. Surg..

[25] Rouzé S., Flécher E., Revest M., Anselmi A., Aymami M., Roisné A., Guihaire J., Verhoye J.P. (2016). Infective Endocarditis with Paravalvular Extension: 35-Year Experience. Ann. Thorac. Surg..

